# MEC-10 and MEC-19 Reduce the Neurotoxicity of the MEC-4(d) DEG/ENaC Channel in *Caenorhabditis elegans*

**DOI:** 10.1534/g3.115.023507

**Published:** 2016-02-17

**Authors:** Yushu Chen, Shashank Bharill, Robert O’Hagan, Ehud Y. Isacoff, Martin Chalfie

**Affiliations:** * Department of Biological Sciences, Columbia University, New York, New York 10027; † Department of Molecular and Cell Biology and Helen Wills Neuroscience Institute, University of California, Berkeley, California 94720; ‡ Department of Genetics, Rutgers, The State University of New Jersey, Piscataway, New Jersey 08854

**Keywords:** DEG/ENaC channels, *Caenorhabditis elegans*, physiological suppressors, touch sensitivity, neurodegeneration

## Abstract

The *Caenorhabditis elegans* DEG/ENaC proteins MEC-4 and MEC-10 transduce gentle touch in the six touch receptor neurons . Gain-of-function mutations of *mec-4* and *mec-4(d)* result in a hyperactive channel and neurodegeneration *in vivo*. Loss of MEC-6, a putative DEG/ENaC-specific chaperone, and of the similar protein POML-1 suppresses the neurodegeneration caused by a *mec-4(d)* mutation. We find that mutation of two genes, *mec-10* and a new gene *mec-19* (previously named C49G9.1), prevents this action of POML-1, allowing the touch receptor neurons to die in *poml-1mec-4(d)* animals. The proteins encoded by these genes normally inhibit *mec-4(d)* neurotoxicity through different mechanisms. MEC-10, a subunit of the mechanosensory transduction channel with MEC-4, inhibits MEC-4(d) activity without affecting MEC-4 expression. In contrast, MEC-19, a membrane protein specific to nematodes, inhibits MEC-4(d) activity and reduces MEC-4 surface expression.

Degenerin and epithelial Na^+^ channel (DEG/ENaC) proteins form sodium-selective, amiloride-sensitive channels in invertebrates and vertebrates. These channels can be constitutively active [the ENaC channels (Lingueglia *et al.* 1993; Canessa *et al.* 1993)], or they can be gated mechanically (O’Hagan *et al.* 2005), by acid (Waldmann *et al.* 1997), or by small peptides [FMRFamide peptide-gated Na^+^ channel (Lingueglia *et al.* 1995)]. DEG/ENaC channels serve a wide range of functions, including mechanosensation (Geffeney *et al.* 2011; O’Hagan *et al.* 2005; Zhong *et al.* 2010), sour and sodium taste (Liu *et al.* 2003; Chandrashekar *et al.* 2010; Wang *et al.* 2008), synaptic plasticity, learning and memory (Wemmie *et al.* 2002; Wemmie *et al.* 2003), and sodium homeostasis (Loffing and Korbmacher 2009; Schild 2010).

Accumulation of high levels of constitutively-open ENaC channels or hyperactivation of gated DEG/ENaC channels can be very detrimental. For example, the excessive accumulation of ENaC channels in the kidney leads to increased sodium reabsorption and hypertension in Liddle syndrome in humans (Shimkets *et al.* 1994; Hansson *et al.* 1995a,b; Goulet *et al.* 1998). The hyperactivation of ASIC1 channels by ischemia and stroke-induced local acidosis causes massive neuronal death in mouse brains (Xiong *et al.* 2004). Gain-of-function mutations affecting *Caenorhabditis elegans (C. elegans)* DEG/ENaC proteins produce hyperactive channels that cause neuronal lysis and degeneration (Shreffler *et al.* 1995; Driscoll and Chalfie 1991; Chalfie and Wolinsky 1990) or hypercontraction of muscle (Park and Horvitz 1986; Liu *et al.* 1996). Studying the molecular mechanisms that regulate hyperactive DEG/ENaCs can better our understanding of both channel hyperactivation-induced toxicity and normal channel physiology.

In *C. elegans*, the DEG/ENaC protein MEC-4 is essential for touch sensitivity (Chalfie and Sulston 1981; Driscoll and Chalfie 1991). Together with another DEG/ENaC protein, MEC-10, MEC-4 forms a trimeric channel that transduces touch in the six touch receptor neurons (TRNs; these cells are the 2 ALM, 2 PLM, 1 AVM, and 1 PVM neurons; Árnadóttir *et al.* 2011; O’Hagan *et al.* 2005; Chen *et al.* 2015). The *mec-4(d)* mutation *e1611* (producing an A713T substitution) results in constitutive channel activation and thus neurodegeneration (Driscoll and Chalfie 1991; Brown *et al.* 2007; Goodman *et al.* 2002). The *mec-4(d)*-induced cell death requires three chaperone-like proteins: MEC-6 (paraoxonase-like protein), CRT-1/calreticulin (calcium binding chaperone), and POML-1 (a MEC-6 and paraoxonase-like protein in *C. elegans*) (Xu *et al.* 2001; Chalfie and Wolinsky 1990; Chen *et al.* 2016).

Here we performed a genetic screen for enhancers of *mec-4(d)*−induced TRNs cell death in *poml-1mec-4(d)* genetic background to identify genes that may normally inhibit *mec-4(d)* and, possibly *mec-4(+)* activity. We found that loss of *mec-10* or *mec-19*, a gene previously named C49G9.1 that encodes a novel TRN membrane protein, enhanced *mec-4(d)* toxicity. Their protein products, MEC-10 and MEC-19, reduced MEC-4(d) activity through different mechanisms. MEC-10(+) reduced MEC-4(d) activity without affecting MEC-4 protein level and localization, presumably by affecting channel activity. In contrast, MEC-19 reduced MEC-4 surface expression while inhibiting MEC-4(d) activity.

## Materials and Methods

### *C. elegans* procedures

Unless otherwise indicated, strains were maintained and studied at 20°C according to Brenner (1974) on the OP50 strain of *Eshcerichia coli*. The strains used in this study are given in Table 1. Strains with the *poml-1(ok2266)*, *mec-10(ok1104)*, *mec-19(ok2504)*, *crt-1(ok948)* mutations were obtained from the Caenorhabditis Genetics Center (CGC). *mec-4d(e1611)*, *mec-4(u45)*, and *mec-6(u450)* have been described previously (Huang and Chalfie 1994; Driscoll and Chalfie 1991; Chalfie and Au 1989). *poml-1(u882)* has been described in Chen *et al.* 2016. *mec-19(u898)* was obtained by ethyl methanesulfonate (EMS) mutagenesis as described in the paragraph to follow. Double or triple mutants were created by standard genetics procedures and verified either phenotypically or by polymerase chain reaction (PCR).

**Table 1:** Strains used in these studies

Strain	Genotype
TU3871	*uIs152(mec-3p*::*tagrfp*); *uIs31(mec-17p*::*gfp)*; *poml-1(ok2266) mec-4(e1611)*
TU3964	*mec-10(ok1104) poml-1(ok2266)*
TU3965	*mec-10(ok1104) poml-1(u882)*
TU3968	*uIs152*; *uIs31*; *mec-10(ok1104) poml-1(ok2266) mec-4(e1611)*
TU3974	*mec-6(u450)*; *uIs152*; *uIs31*; *mec-10(ok1104) mec-4(e1611)*
TU4243	*uEx851*(*mec-4p*::*mec-4*::*tagrfp*); *mec-19*(*u898*); *poml-1(ok2266)*
TU4270	*mec-19(ok2504)*; *uIs152*; *uIs31*; *poml-1(ok2266) mec-4(e1611)*
TU4271	*mec-6(u450) mec-19(u898)*; *uIs152*; *uIs31*; *mec-4(e1611)*
TU4327	*mec-19*(*u898)*; *uIs31*; *poml-1(ok2266)*
TU4328	*mec-19*(*u898)*; *uIs31*
TU4355	*mec-19*(*u898)*; *uIs146*(*mec-4p*::*mec-4*::*tagrfp)*
TU4426	*mec-19(u898)*; *uIs31*; *crt-1(ok948)*; *mec-4(e1611)*
TU4735	*uIs31*; *crt-1(ok948)*; *mec-10(ok1104) mec-4(e1611)*

EMS mutagenesis was performed according to Brenner (1974) to identify suppressors of the *poml-1* suppression of *mec-4(d)* degeneration. We mutagenized TU3871 [*uIs152* (*mec-3p*::*tagrfp*); *uIs31(mec-17p*::*gfp)*; *poml-1(ok2266) mec-4d(e1611)*] animals, plated individual P0 animals, and screened their F2 progeny for animals missing red fluorescent protein (RFP) and green fluorescent protein (GFP) in the TRNs but expressing RFP in the FLP neurons, which express *mec-3* but not *mec-17*. Normally in TU3871 animals *mec-3p*::TagRFP labels both the TRNs and the FLP neurons and *mec-17p*::GFP labels only the TRNs.

**Figure 1:**
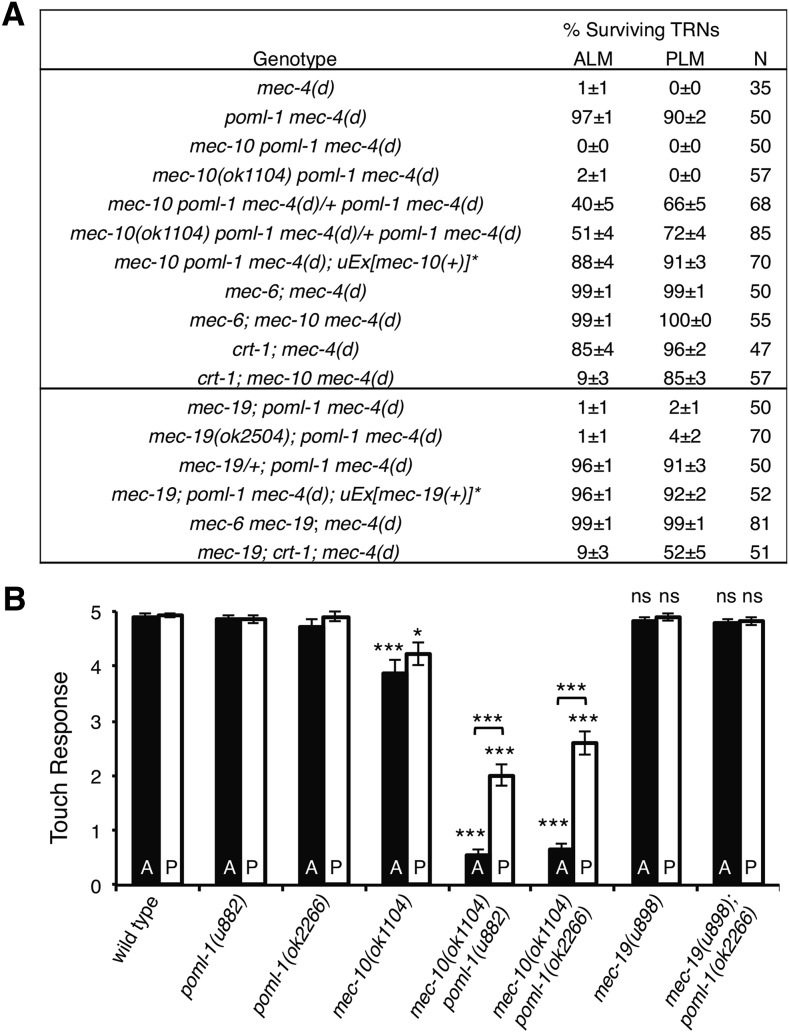
Effect of *mec-10* and *mec-19* mutations on *mec-4(d)* degeneration and touch sensitivity. (A) Loss of *mec-10* and *mec-19*−enhanced touch receptor neurons degeneration in *poml-1 mec-4(d)* animals. N indicates the number of animals examined. All experiments used *poml-1(ok2266)*, *mec-4d(e1611)*, *mec-10(u883)*, *mec-6(u450)*, *crt-1(ok948)*, and *mec-19*(*u898)* unless noted. *mec-10 rescue was examined in four stable lines; *mec-19* rescue was examined in three stable lines. (B) The effect of *mec-10* and *mec-19* mutations on touch sensitivity with or without a *poml-1* mutation (mean ± SEM, n = 30 animals). A = anterior response to 5 touches; *P* = posterior response to 5 touches. The anterior or posterior responses of mutants of *mec-10*, *mec-19*, *mec-10 poml-1*, or *mec-19*; *poml-1* were compared with those of wild-type animals by the Student’s *t*-test with the Bonferroni correction: ***P < 0.001 (raw *P* < 0.0001), *P < 0.05 (raw *P* = 0.0028), ns, not significant. The differences between *mec-10 poml-1* double mutants and a single mutant of *mec-10* or *poml-1* also were significant at *P* < 0.001 (raw *P* < 0.0001) by the Student’s *t*-test with the Bonferroni correction.

Seventeen viable mutants were obtained after screening F2 progeny representing 20,000 haploid genomes. To identify the causal mutations in these mutants, we extracted genomic DNA from the unmutagenized starting strain (TU3871) and 10× outcrossed strain carrying the two complementing autosomal mutations and unoutcrossed strains with two of the 15 X-linked mutations that failed to complement each other using the Gentra Puregene Kit (QIAGEN, Valencia, CA). Whole-genome resequencing was performed by the laboratory of Oliver Hobert (Zuryn *et al.* 2010; Minevich *et al.* 2012). Potential mutations were verified by rescuing the touch cell death phenotype with multiple copies of the wild-type gene (Figure 1A). The remaining X-linked mutations were confirmed as alleles of *mec-10* by sequencing *mec-10* DNA amplified from mutant worms by PCR.

We assayed for gentle touch sensitivity in blind tests as described (Chalfie and Sulston 1981). We quantified the response by counting the number of responses to a total of 10 touches delivered alternately near the head and tail in 30 young adult animals (Hobert *et al.* 1999). We performed *in vivo* electrophysiology as described previously (O’Hagan *et al.* 2005).

### Plasmids and microinjection

*mec-19*::*gfp* (Topalidou and Chalfie 2011) and *mec-4*::*tagrfp* (TU#1175; Chen *et al.* 2015) have been described previously. *myo-2p*::*mCherry* (PCFJ90) was obtained from Addgene (www.addgene.org). *mec-4p*::*aman-2*::*tagrfp* (TU#1181) was made using the Three-Fragment Vector Construction Kit (Invitrogen, Carlsbad, CA). *mec-4* promoter and start codon of 1023 bp was cloned into pDONRP4P1R. *aman-2* coding sequence of 300 bp (Rolls *et al.* 2002) was cloned into pDONR221. *tagrfp* with a *unc-54* 3′UTR was cloned into pDONRP2RP3.

We microinjected 10 ng/μL *mec-19*::*gfp* and 5 ng/μL *aman-2*::*tagrfp*, 2 ng/μL *myo-2p*::*mCherry* (PCFJ90) and 40 ng/μL of the *lin-15*(+) plasmid, and pBluescript SK plasmid to make up to 100 ng/μL DNA in total. For rescue experiments, we injected 2 ng/μL PCR product of *mec-10* or *mec-19*, 2 ng/μL *inx-20p*::*gfp* linearized by *Sph*I, and 125 ng/μL genomic DNA linearized by *Eco*RI and *Kpn*I from OP50 *E. coli*.

### Microscopy and immunofluorescence

Fluorescence and immunofluorescence were observed with a Zeiss Axio Observer Z1 inverted microscope equipped with 63× and 100×, NA 1.40 oil immersion objectives and a Photometrics CoolSnap HQ^2^ camera (Photometrics, Tucson, AZ). Confocal images were acquired using Confocal ZEISS LSM700 equipped with a 63× NA 1.40 oil immersion objective. Live animals were anesthetized using 0.1 mM 2, 3-butanedione monoxime in 10 mM HEPES, pH 7.4.

Immunostaining was performed according to Miller and Shakes (1995) using a mouse antibody against MEC-4 (ab22184, Abcam, Cambridge, MA) diluted 1:200 and an Alexa Fluor 488-conjugated goat anti-mouse antibody (Life Technologies, Carlsbad, CA) diluted 1:700.

MEC-4::TagRFP or immunofluorescence intensity in the cell body was determined by measuring the mean intensity of the entire cell body (20−30 μm^2^) and subtracting the mean intensity of nearby background of the same size using Image J (rsbweb.nih.gov/ij/). The intensity of the MEC-4::TagRFP puncta in TRN neurites was measured using the Puncta Analysis Toolkit beta developed by Dr. Mei Zhen (Samuel Lunenfeld Research Institute, Toronto, Canada). Puncta were examined over a region equivalent to approximate ten cell body lengths (~50 μm) starting near the cell bodies. The intensity of MEC-4 immunofluorescence in the TRN neurite was determined by measuring the mean intensity of 30−50 μm lengths of the PLM neurite between cell bodies of PLM and PVM using Image J. We performed single-molecule fluorescence *in situ* hybridization as described previously (Topalidou *et al.* 2011).

### Oocyte experiments

cRNA expression and electrophysiology in *Xenopus laevis* oocytes followed the procedures and used the plasmids described in Goodman *et al.* (2002) except for the experiments with CaV2.1, which followed Fan *et al.* (2012). *mec-19* cDNA of 390 bp was obtained by reverse-transcription PCR from cDNA library (generated by reverse-transcription using wild-type mRNA) and was cloned in pGEM-HE (Liman *et al.* 1992). A total of 10 ng cRNA of *mec-4(d)*, *mec-2*, and *mec-10*; 1 ng *mec-6*; and 1 ng cRNA of *mec-19* were injected to oocytes unless noted (oocytes were a gift of Dr. Jian Yang and were obtained from frogs from Xenopus I, Dexter, MI, or Nasco, Fort Atkinson, WI). Oocytes were maintained as described previously (Árnadóttir *et al.* 2011). Membrane current was measured 4−6 d after RNA injection using a two-electrode voltage clamp as described previously (Goodman *et al.* 2002).

Immunoprecipitation of C-terminally HA-tagged MEC-19 and N-terminally Myc-tagged MEC-4(d) were performed 5−6 d after cRNA injection as described previously (Goodman *et al.* 2002) by using a rabbit polyclonal antibody against the HA tag (sc-805; Santa Cruz Biotechnology, Dallas, TX) and Protein A/G PLUS-Agarose (Santa Cruz Biotechnology). Protein was detected by using mouse monoclonal antibodies against the Myc (9E10; Sigma-Aldrich, St. Louis, MO) and the HA (sc-7392; Santa Cruz Biotechnology) tags and horseradish peroxidase−conjugated secondary antibodies (Jackson ImmunoResearch Laboratories, West Grove, PA). Approximately three oocytes equivalents were loaded for the immunoprecipitation, and total lysate of one oocyte were loaded for the input. The specificity of the immunoprecipitation was confirmed in three ways. First, EGFP::HA, a negative control generated by the injection of 1 ng of the encoding cRNA, did not immunoprecipitate Myc::MEC-4(d). Second, MEC-19::HA did not immunoprecipitate Myc::EGFP when 1 ng cRNA of constructs encoding each were coinjected. Third, the oocyte membrane protein β-integrin was not detected in the immunocomplexes by a monoclonal antibody against it (8C8; Developmental Studies Hybridoma Bank, University of Iowa, IA).

Imaging and stoichiometry analysis of protein complexes on oocyte membranes using total internal reflection fluorescence microscopy were performed 1−2 d after cRNA injection as described previously (Ulbrich and Isacoff 2008, 2007; Abuin *et al.* 2011). The constructs of N and C-terminally EGFP-tagged MEC-4 have been described in Chen *et al.* (2015).

### Statistics

Statistical analysis was performed using the Student’s *t*-test, one-way analysis of variance (ANOVA), one sample *t*-test or the Mann−Whitney *U*-test using GraphPad Prism 5 software (http://www.graphpad.com/scientific-software/prism/) unless otherwise noted. The Student’s *t*-test was used for most of the experiments, with the Welch’s correction when data being compared did not have equal variances. The Mann−Whitney *U*-test was used to analyze the number of MEC-4 spots on the surface of *Xenopus* oocytes. *P*-values were adjusted with a Bonferroni correction when multiple comparisons were performed, and the raw *P*-values were also provided. The one sample *t*-test was used to analyze the western blots of MEC-4 expression in total lysates of *Xenopus* oocytes. One-way ANOVA was used to compare the number of mRNA molecules in wild type and two *mec-19* mutants. In all figures, *, **, *** indicate Bonferroni-corrected *P*-values of < 0.05, < 0.01, and < 0.001, respectively; ns, not significant.

### Data and reagent availability

All strains used and/or generated in this study are available upon request. Strains are given in Table 1 and Table 2.

**Table 2:** *poml-1* suppression of *mec-4(d)* requires *mec-10* and *mec-19*

Gene	Allele	Mutation	D/R	% ALM	% PLM
*mec-10*	*u883*	TGG > TGA, 95W > Stop	Semi-D	0	0
	*u884*	CAG > TAG, 147Q > Stop	Semi-D	0	4
	*u885*	TGG > TGA, 618W > Stop	R	0	2
	*u886*	TGC > TAC, 170C > Y	R	0	3
	*u887*	TCC > TTC, 471S > F	R	2	12
	*u888*	CGC > TGC, 507R > C	R	1	6
	*u889*	TGC > TAC, 557C > Y	R	2	13
	*u890*	GTG > ATG, 573V > M	R	5	17
	*u891*	G > A splicing junction, exon 2 - intron 2	R	1	5
	*u892*	G > A splicing junction, exon 6 - intron 6	R	2	11
	*u893*	A > T the 3rd nucleotide, intron 6	R	2	8
	*u894*	G > A splicing junction, exon 9 - intron 9	Semi-D	2	2
	*u895*	G > A splicing junction, exon 14 - intron 14	Semi-D	1	4
	*u896*	G > A, the 5th nucleotide, intron 16	R	2	1
	*u897*	Deletion*^a^*	Semi-D	6	18
*mec-19*	*u898*	Deletion of the first exon	R	1	2
*mec-3*	*u899*	T > A, the 5th last nucleotide, intron 2 of isoform a	R	0	1

D, dominant; R, recessive.

a DNA from *u897* animals could not be amplified using primers that were 120 bp upstream of the start ATG and 80 bp downstream of the stop codon. n = 50 animals.

## Results

### Loss of *mec-10* or *mec-19* enhances TRN cell death in *poml-1 mec-4(d)* animals

Loss of *poml-1* (*e.g.*, with the *ok2266* mutation) lowers MEC-4 protein levels and suppresses *mec-4(d)*−induced TRN degeneration (90% of the TRNs live; Chen *et al.* 2016). To identify genes whose products normally reduce MEC-4 activity and hence increase the TRN cell death when mutated, we screened for mutations that increased TRN cell death in *poml-1(ok2266) mec-4(d)* animals. The starting strain (TU3871) also contained *mec-3p*::*tagrfp* to label the TRNs and the FLP neurons and *mec-17p*::*gfp* to label the TRNs. Mutations that allowed TRN deaths would lack the TRN label but not the FLP label.

Seventeen such mutations were found among F2 progeny representing 20,000 haploid genomes after EMS mutagenesis [Table 2; one mutation was a *mec-3* non-coding mutation, which gave the phenotype by causing *mec-3* expression in the FLP neurons, but not in the TRNs]. Fifteen of the mutations were X-linked and failed to complement each other. All 15 strains had *mec-10* mutations; these mutations included nonsense alleles, missense alleles, a deletion allele, and several splice junction alleles. Several of these *mec-10* mutations acted semidominantly. The *mec-10(ok1104)* allele, which is considered to be a loss-of-function deletion (Árnadóttir *et al.* 2011), also enhanced the TRN cell death in *poml-1(ok2266) mec-4(d)* animals semidominantly (Figure 1A). Addition of the wild-type gene rescued the effects of the *mec-10* mutations (Figure 1A). The inhibitory effect of MEC-10 on MEC-4(d)−induced TRN neurodegeneration is consistent with our previous finding that MEC-10 decreased MEC-4(d) activity in *Xenopus* oocytes (Goodman *et al.* 2002). Thus, both the *in vivo* and *in vitro* data suggest that MEC-10(+) inhibits MEC-4(d) channel activity.

The remaining mutation deleted a 288-bp sequence containing 19 bp upstream of start codon, the first exon and part of the first intron from C49G9.1. This mutation enhanced the *mec-4(d)* phenotype recessively (Figure 1A). The effect on *mec-4(d)* degeneration was caused by this mutation, because it could be rescued by the wild-type gene (Figure 1A). Given that a larger deletion allele (*ok2504*) gave a similar phenotype, both mutations are likely to be null alleles (Figure 1A). Because of its effect on touch-sensitivity in a sensitized background (see MEC-19 reduces MEC-4 expression in the TRNs), we have renamed the gene *mec-19*.

We also tested the effect of the *mec-10* and *mec-19* mutations on the suppression of *mec-4(d)* by *crt-1* and *mec-6* mutations, which are known to suppress *mec-4(d)* deaths (Chalfie and Wolinsky 1990; Xu *et al.* 2001). (Both CRT-1 and MEC-6 act as endoplasmic reticulum chaperones for the production of MEC-4; Chen *et al.* 2016). Loss of *mec-10* and *mec-19* enhanced cell death in *crt-1*; *mec-4(d)* animals, but to a lesser extent (Figure 1A) than they did in the *poml-1* animals. In contrast, neither *mec-10* nor *mec-19* mutations promoted *mec-4(d)* degeneration when *mec-6* gene was absent (Figure 1A), probably due to a broader role of *mec-6* in *mec-4(d)* function.

We next tested the effect of *mec-10* or *mec-19* mutations on touch sensitivity with or without the *poml-1* mutation. The *mec-10* null allele *ok1104* caused a modest loss of the touch sensitivity (as previously seen by Árnadóttir *et al.* 2011), which was further reduced by *poml-1* null mutations (*ok2266* and *u882*; Figure 1B). The *mec-10poml-1* double mutation had a stronger effect on anterior touch sensitivity than posterior touch sensitivity (Figure 1B). These data suggest that MEC-10 and POML-1 act additively in touch sensitivity but against each other with regard to MEC-4(d) channel activity. In contrast to *mec-10*, loss of *mec-19* did not detectably change touch sensitivity either with or without a *poml-1* mutation (Figure 1B).

### MEC-19 reduces MEC-4 expression in the TRNs

**Figure 2:**
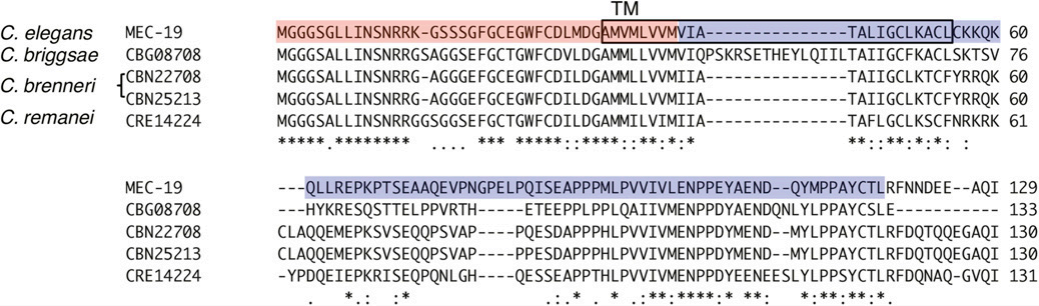
The amino acid sequence of MEC-19 and its homologs in other nematode species. The predicted transmembrane (TM) region is in the black box. Sequence alignment was performed using ClustalW2 (http://www.ebi.ac.uk/Tools/msa/clustalw2/). The sequences deleted in *mec-19(u898)* and *mec-19(ok2504)* are highlighted in red and blue, respectively.

**Figure 3:**
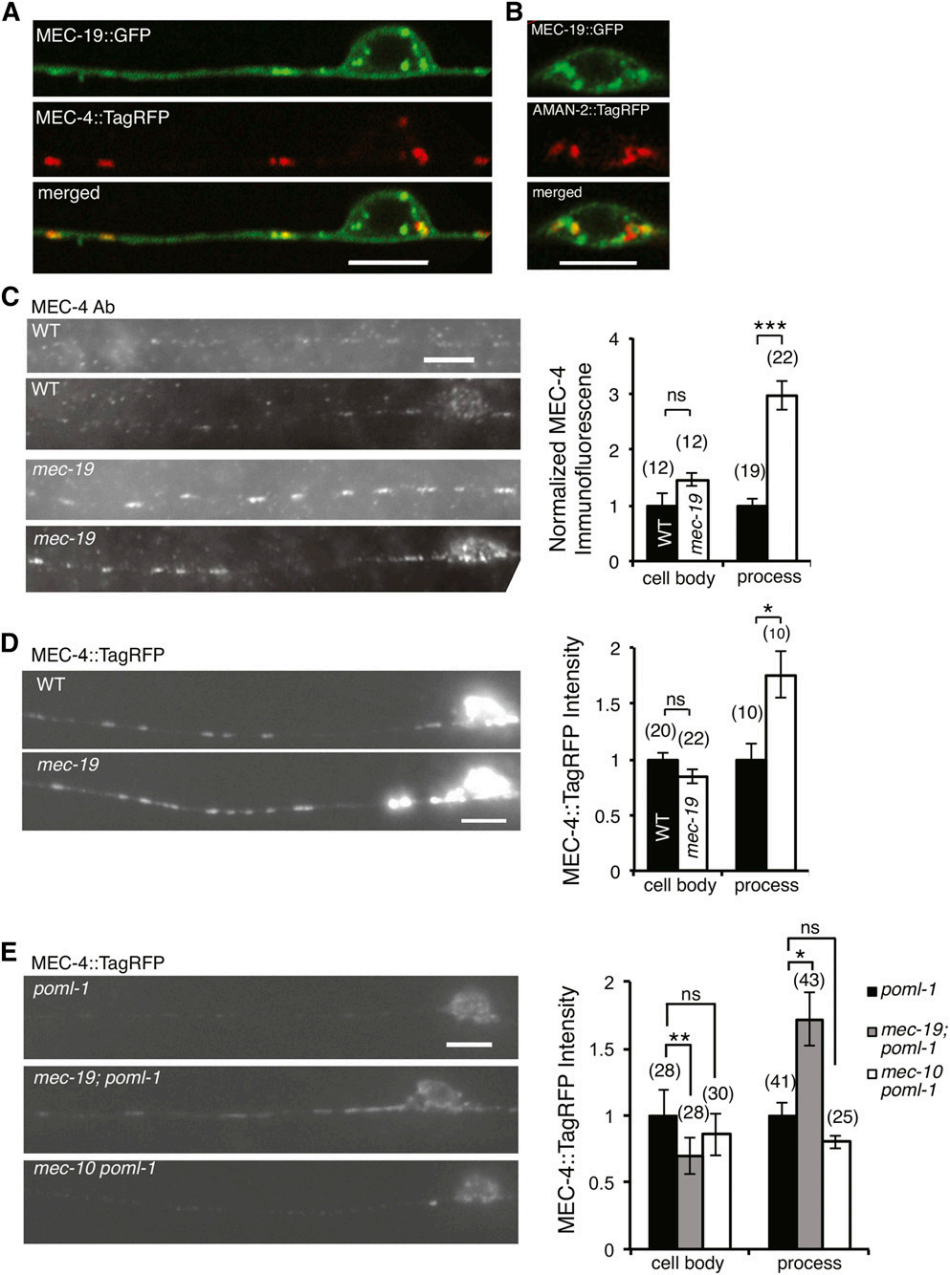
MEC-19 expression pattern and the effect of *mec-19* mutation on the amount of MEC-4 in TRNs. (A, B) Confocal images showing the partial overlap of MEC-19::GFP with MEC-4::TagRFP in cell body and proximal neurite (A) and the Golgi marker (AMAN-2::TagRFP) in the cell body (B). Scale bar = 5 μm (here and in C, D, and E). (C) Images (left panel) and quantification (right panel, mean ± SEM) of MEC-4 labeling with an anti-MEC-4 antibody in the touch receptor neurons (TRNs) of wild type (WT) animals and *mec-19*(*u898*) mutants. Each pair of panels on the left shows the TRN neurite (upper) and cell body (lower). Immunofluorescence intensity was normalized and compared with that of the wild type. The number of PLM neurons examined is given in parentheses (here and in D and E). ***P < 0.001 (raw *P* < 0.0001), Student’s *t*-test with the Bonferroni correction. *mec-19* loss did not change the density of MEC-4 puncta (puncta/μm of the TRN neurite): 0.24 ± 0.01 for wild type *vs.* 0.24 ± 0.01 for *mec-19* (mean ± SEM, not significant by Student’s *t*-test here and in D and E). (D) Images and quantification (mean ± SEM) of MEC-4::TagRFP in the TRN of wild-type (WT) animals and *mec-19*(*u898*) mutants. MEC-4::TagRFP fluorescence intensity was normalized and compared with that of the wild type. *P < 0.05 (raw *P* = 0.01), ns, not significant, Student’s *t*-test with the Bonferroni correction. *mec-19* loss did not change the density of MEC-4::TagRFP puncta: 0.26 ± 0.02 for wild-type *vs.* 0.26 ± 0.02 for *mec-19*. (E) Images (left panel) and quantification of MEC-4::TagRFP fluorescence intensity (mean ± SEM) in TRNs of *poml-1(ok2266)*, *mec-19(u898)*; *poml-1(ok2266)* or *mec-10(ok1104) poml-1(ok2266)* animals. Images of (D) and (E) were taken and processed under the same conditions and can, thus, be compared. Fluorescence intensity was normalized and compared with that of *poml-1*. **P < 0.01 (raw *P* < 0.001), *P < 0.05 (raw *P* < 0.005), ns, not significant, Student’s *t*-test with the Bonferroni correction. The density of MEC-4::TagRFP puncta in the first 50-60 µm of the TRN neurite starting from the cell body was not different between *poml-1* (0.22 ± 0.02) and *mec-19*; *poml-1* (0.23 ± 0.01).

*mec-19* encodes a novel membrane protein of 129 amino acids with one predicted transmembrane domain near its N-terminus (Figure 2). We identified similar proteins in other nematodes but not in other organisms (Figure 2). The gene is expressed in the TRNs, FLP neurons, and PVD neurons (Topalidou and Chalfie 2011). A MEC-19::GFP translational fusion was found throughout the TRN neurite and also on the plasma membrane and spots within the TRN cell body (Figure 3, A and B); its expression overlapped only partially with MEC-4 (Figure 3A) and MEC-2 (Topalidou and Chalfie 2011) in the proximal neurite and cell body. In the cell body, MEC-19 spots also were found to partially overlap with the Golgi marker AMAN-2::TagRFP (Figure 3B).

Loss of *mec-19* increased the amount of MEC-4 in the TRN neurite as measured by the use of an anti-MEC-4 antibody (Figure 3C) and MEC-4::TagRFP fusion protein (Figure 3D). Moreover, loss of *mec-19* increased MEC-4::TagRFP fluorescence in the TRN neurites by 70% in *poml-1* mutants (Figure 3E). *mec-19*; *poml-1* double mutants also expressed 30% less MEC-4 in their cell bodies than *poml-1* mutants (Figure 3E), but a similar effect was not observed in wild type (Figure 3, C and D). In contrast, loss of *mec-10* did not increase MEC-4::TagRFP levels either in *poml-1* mutants (Figure 3E) or in wild-type animals (Árnadóttir *et al.* 2011). The increased MEC-4 was not due to an increase in the amount of steady state *mec-4* mRNA as measured by single-molecule fluorescence *in situ* hybridization (8.2 ± 0.3 mRNA molecules/PLM for *mec-19*(*u898*), 8.6 ± 0.3 for *mec-19*(*ok2504*), and 8.7 ± 0.4 for wild type, mean ± SEM, n = 20, not significant by one-way ANOVA).

Thus, MEC-19 affects the amount of MEC-4 in the TRN neurite. The increase in cell death in *mec-19*; *poml-1mec-4(d)* animals was likely due, at least in part, to elevated levels of surface MEC-4(d). In contrast, *mec-10* did not appear to affect MEC-4 protein levels and presumably enhanced *mec-4(d)* cell deaths through a different mechanism.

**Figure 4:**
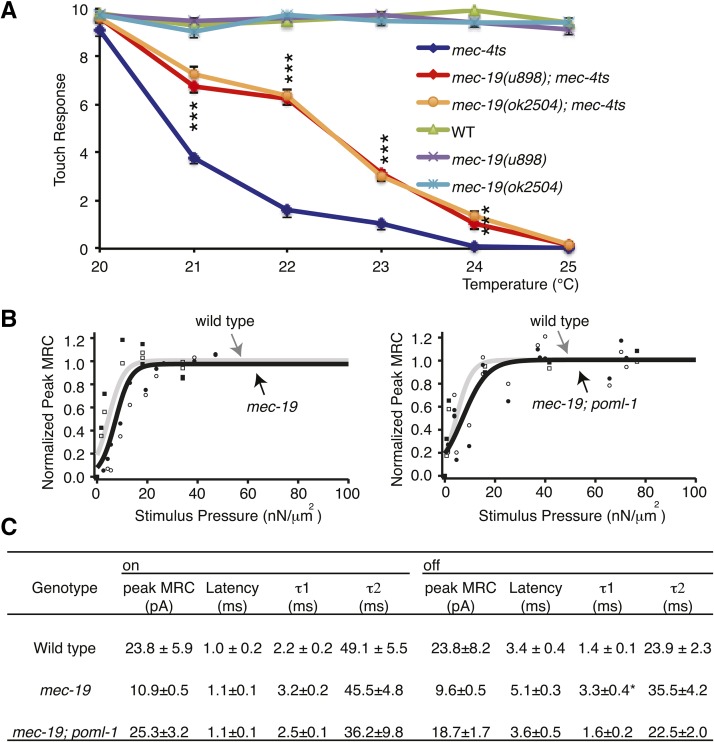
The effect of *mec-19* mutations on touch sensitivity and on the mechanoreceptor current (MRC) *in vivo*. (A) *mec-19*(*u898)* and *mec-19(ok2504*) increase touch sensitivity of *mec-4ts(u45)* animals (mean ± SEM, n = 30). Difference of touch responses between *mec-4ts* and *mec-19(u898)*; *mec-4ts* or *mec-19(ok2504)*; *mec-4ts* at 21°, 22°, 23°, and 24°; all had Bonferroni-corrected *P* < 0.001 (raw *P* < 0.0001) by the Student’s *t*-test, whereas the difference at 20° and 25° was not significant by the Student’s *t*-test. Touch response between *mec-19(u898)*; *mec-4ts* and *mec-19(ok2504)*;*mec-4ts* was not significantly different from 20° to 25° by the Student’s *t*-test. (B) *mec-19(u898)* did not produce significant changes in the current *vs.* pressure (I *vs.* P) relation of MRCs. The peak amplitude of MRCs recorded from PLM (at -74 mV) at the onset of a mechanical stimulus was normalized to the maximum MRC current. Wild type is represented by the gray curve and white symbols. Each symbol (rectangle or circle) represents a recording from a different PLM cell. *mec-19* or *mec-19*; *poml-1* is represented by the black curve and black symbols. Wild type: P_1/2_ = 4.5 ± 0.7 nN/μm^2^, P_slope_ = 3.1 ± 0.7, N = 3 (Chen *et al.* 2016). *mec-19*: P_1/2_ = 7.3 ± 0.9 nN/μm^2^, P_slope_ = 3.0 ± 0.6, N = 2. *mec-19*; *poml-1*: P_1/2_ = 7.0 ± 1.2 nN/μm^2^, P_slope_ = 5.0 ± 1.0, N = 2. Data are represented as mean ± SD. N indicates the number of cells tested. (C) *mec-19* mutation had little effect on the average peak MRC amplitude, latency, activation (τ1), and adaptation (τ2) calculated from MRC response at the onset and offset of mechanical stimuli (mean ± SEM). The data of wild type are from Chen *et al.* 2016. *P < 0.05, compared to the wild-type and *mec-19*; *poml-1* double mutants, one-way analysis of variance with Tukey *post hoc*.

Consistent with the increased amount of MEC-4 in *mec-19* TRN neurites, *mec-19* loss increased the touch sensitivity of *mec-4 ts* animals (Gu *et al.* 1996) at various temperatures (Figure 4A). However, loss of *mec-19* did not detectably affect touch sensitivity in wild-type or *poml-1* mutants (Figure 4A and Figure 1B) and had only modest effects on the response of the mechanoreceptor current to different pressures, the peak amplitude at saturating stimuli, and the kinetics of the mechanoreceptor current (Figure 4, B and C).

### MEC-19 reduces MEC-4 surface expression and activity in *Xenopus* oocytes

**Figure 5:**
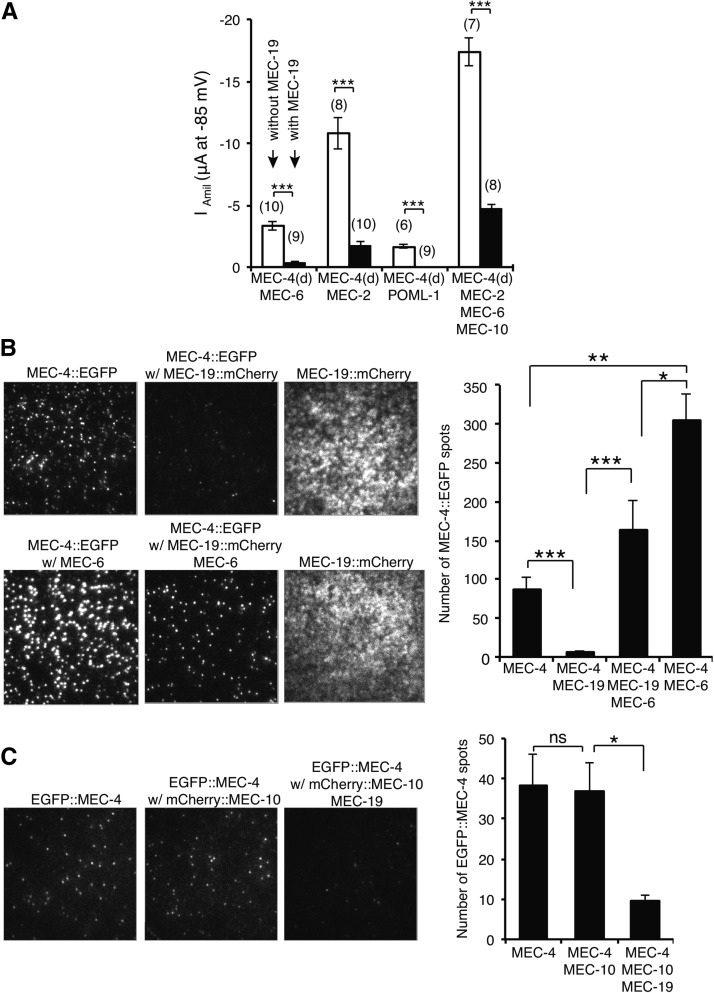
The effect of MEC-19 on MEC-4(d) activity and MEC-4 surface expression in *Xenopus* oocytes. (A) The effect of MEC-19 on the MEC-4(d) amiloride-sensitive current (mean ± SEM) in the presence of MEC-6, MEC-2, POML-1, or MEC-10 in oocytes. The number of tested oocytes from two individual frogs is given in parentheses. ***P < 0.001 (raw *P* < 0.0001 for data with MEC-6, POML-1 and MEC-6/MEC-2/MEC-10, raw *P* = 0.0002 for data with MEC-2), Student’s *t*-test with the Bonferroni correction. (B) Images (left panel) and quantification (right panel) of C-terminally EGFP-tagged MEC-4 fluorescent spots by total internal reflection fluorescence (TIRF) imaging in the presence of MEC-19 and MEC-6 (mean ± SEM, n = 8-15 patches from 7-10 cells of two different batches. 10 ng cRNA for MEC-4::EGFP, 1 ng cRNA for MEC-6, and 0.5 ng cRNA for MEC-19 were injected to oocytes. Statistics were determined by Mann−Whitney *U*-test with the Bonferroni correction. Raw *P*-values, *P = 0.005, **P = 0.0004, ***P < 0.0001. (C) Images (left panel) and quantification (right panel) of N-terminally EGFP-tagged MEC-4 spots by TIRF imaging in the presence of MEC-19 and MEC-10 (mean ± SEM, n = 9-12 patches from 7-10 cells). 2.5 ng cRNA for EGFP::MEC-4 and mCherry::MEC-10, 1 ng cRNA for MEC-19 were injected to oocytes. *P < 0.05 by Mann−Whitney *U*-test with the Bonferroni correction (raw *P* = 0.009).

**Figure 6:**
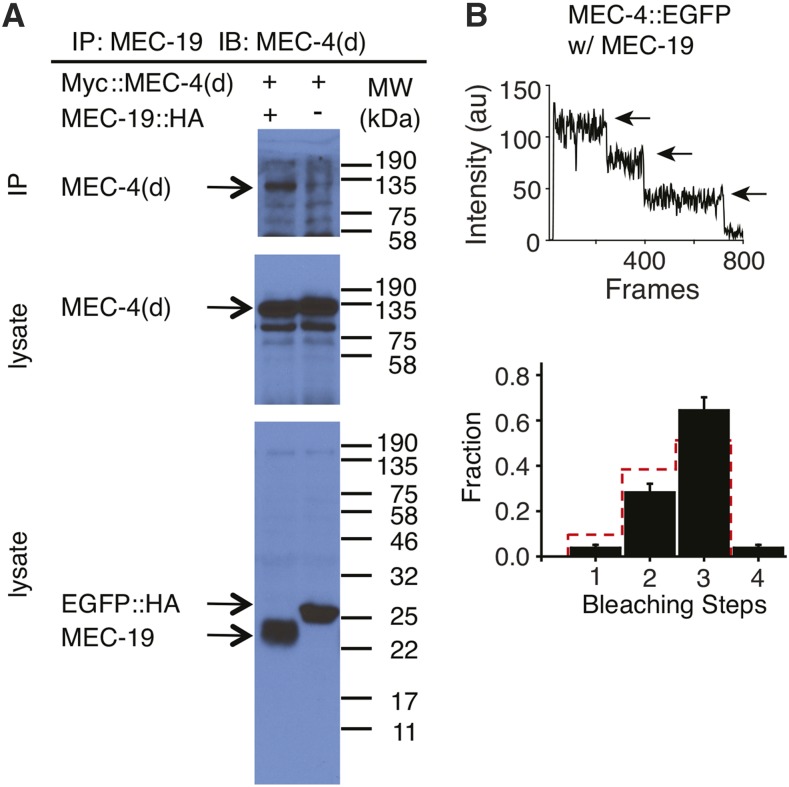
Physical interactions between MEC-4 and MEC-19 expressed in *Xenopus* oocytes. (A) Immunoprecipitation (IP) of Myc::MEC-4(d) by MEC-19::HA. IB = immunoblot probe. MEC-19::HA reduced the MEC-4(d) current amplitude when coexpressed with MEC-6 as untagged proteins [I _Amil_ (at −85 mV) = −0.12 ± 0.10 μA, mean ± SEM, n = 9]. The negative control (−) is HA-tagged EGFP. Molecular weights (kDa) of the protein markers used in the experiments are indicated on the right. (B) An example (upper panel) and quantification (lower panel) of the photobleaching of MEC-4::EGFP trimers in the presence of MEC-19 on oocyte surface. The observed frequency distribution of the number of bleaching steps (black bars) and the predicted binomial distribution for trimers (red dotted bars) are indicated. The error bars in the subunit counting data show counting errors and are given by I/N*√n (n = total number of spots for each step; N = total number of spots for all steps).

We next tested the effect of MEC-19 on MEC-4(d) currents in *Xenopus* oocytes. MEC-19 dramatically reduced the amiloride-sensitive current of MEC-4(d) coexpressed with MEC-6, POML-1, MEC-2, or MEC-10 by approximately 70–80% (Figure 5A). [MEC-19 alone produced an amiloride-resistant current when expressed at a greater concentration in oocytes: I (at −85 mV) = −2.5 ± 0.4 μA (mean ± SEM) for 2.5 ng cRNA *vs.* I = −0.2 ± 0.2 μA (n = 4) for 1 ng cRNA for oocytes 5 d after injection.] Thus, both *in vivo* and *in vitro* experiments suggest that wild-type MEC-19 inhibits MEC-4(d) channel activity. Part or all of this inhibition likely resulted from the loss of surface MEC-4 in oocytes, which was seen with total internal reflection fluorescence microscopy (Figure 5, B and C). MEC-19 reduced MEC-4 surface expression with or without MEC-10 (Figure 5, B and C; MEC-10 did not affect MEC-4 surface expression). Even in the presence of MEC-6, MEC-19 still reduced MEC-4 surface expression by nearly 50% (Figure 5B). The reduced MEC-4 surface expression in the presence of MEC-19 was not due to generally poor surface expression, because MEC-19 was well expressed on the surface of oocytes (Figure 5B). The reduced MEC-4 surface expression also was not due to a reduction in total MEC-4 protein level in oocytes (relative amount was 1 without MEC-19 *vs.* 0.99 ± 0.02 with MEC-19, mean ± SEM, n = 3 independent experiments, not significant by one sample *t*-test). The action of MEC-19 on MEC-4(d) could be due to its physical interaction with it, since C-terminally HA-tagged MEC-19 coimmunoprecipitated with N-terminally Myc-tagged MEC-4(d) in oocytes (Figure 6A).

MEC-19 affected at least one other membrane channel, since it largely reduced the current from the human P/Q-type calcium channel CaV2.1 in frog oocytes (the maximal current of CaV2.1 was −6.3 ± 1.1 µA without MEC-19 *vs.* −0.7 ± 0.2 µA with MEC-19, mean ± SEM, n = 5, *P* < 0.01, Student’s *t*-test). MEC-19, however, did not affect channel proteins generally, since the surface expression of the BEST1 chloride channel (Sun *et al.* 2002) was unchanged in oocytes (the number of EGFP::BEST1 fluorescent spots on the surface was 99 ± 21 without MEC-19 and 162 ± 26 with MEC-19, mean ± SEM, n = 15 patches from 7-8 cells, not significant by Student’s *t*-test).

Because the expression of MEC-19 overlapped with that of MEC-4 and MEC-2 in the TRNs and coimmunoprecipitated with MEC-4(d) in oocytes, we asked whether it was part of the MEC-4/MEC-10 channel. We tagged MEC-19 with EGFP/mCherry at its C termini and expressed them in oocytes. The tagged protein retained its normal function because it acted like the untagged protein in rescuing the *mec-19* enhancement of TRN cell death in *poml-1mec-4(d)* animals (surviving TRNs, ALM 94 ± 2%, PLM 92 ± 3%, mean ± SEM, n = 40 from five stable lines), and reduced the MEC-4(d) current amplitude when coexpressed with MEC-6 in oocytes [I _Amil_ (at −85 mV) = −0.17 ± 0.07 μA, mean ± SEM, n = 4]. The stoichiometry of MEC-19 could not be determined because the molecules moved on the surface of oocytes even in the presence of MEC-4, and they did not colocalize with MEC-4 (Supporting Information, File S1). In addition, MEC-19 did not change the stoichiometry of the MEC-4 trimer (Chen *et al.* 2015) on the oocyte surface (Figure 6B), an indication that this protein is not incorporated into the MEC-4 channel complex.

## Discussion

The *poml-1mec-4(d)* double mutant provides a sensitized background in which to screen for genes that normally inhibit *mec-4(d)* degeneration. Using this double mutant, we identified two inhibitors, MEC-10 and MEC-19, that function downstream of POML-1. The average mutation rate in *C. elegans* for EMS mutageneses is approximately 1 in 2000 haploid genomes (Brenner 1974; Greenwald and Horvitz 1980). By examining the animals representing 20,000 haploid genomes, we are, thus, likely to have saturated for genes whose loss causes TRN degeneration in the *poml-1mec-4(d)* background. The number of *mec-10* alleles (15) supports this conclusion. The *mec-10* alleles we found had a variety of defects, including missense, nonsense, and deletion mutations. In contrast, our previous screens for touch insensitive mutants only resulted in *mec-10* missense mutations (Huang and Chalfie 1994). In fact animals lacking MEC-10 retain considerable touch sensitivity, a result that suggested that MEC-10 was partially redundant for touch sensitivity (Árnadóttir *et al.* 2011). The present screen, however, revealed a role for MEC-10 in the control of the MEC-4 channel.

The role for MEC-10 remains, however, elusive, because MEC-10 seems to have opposite effects on MEC-4 and MEC-4(d) channels. MEC-10 is needed for the optimal activity of the MEC-4 mechanotransduction channel, because its loss *in vivo* decreases the mechanoreceptor current amplitude by 25% and modestly decreases touch sensitivity (Árnadóttir *et al.* 2011). In contrast, MEC-10 inhibits MEC-4(d) both *in vivo* and *in vitro*: MEC-10 loss increases *mec-4(d)* toxicity in *poml-1* mutants, and MEC-10 decreases the macroscopic MEC-4(d) current amplitude carried by either Na^+^ or Ca^2+^ in *Xenopus* oocytes (Goodman *et al.* 2002; Bianchi *et al.* 2004). These differences may result because the MEC-4 and MEC-4(d) channels function differently. Specifically, the wild-type MEC-4 channel may need MEC-10 to allow it to be maximally gated, whereas the MEC-4(d) channel, which is constitutively open, allows more current when MEC-10 is absent. Because MEC-10 does not affect MEC-4(d) surface expression (Árnadóttir *et al.* 2011), single-channel conductance, or open probability (Brown *et al.* 2008) in oocytes, it may act by inactivating some MEC-4(d) channels, making them unable to be opened.

In contrast to yielding many independent *mec-10* mutants, our screen gave a single *mec-19* strain, albeit one that contained an early deletion within the gene. The small size of the gene (MEC-19 has only 129 amino acids) is a likely explanation for the dearth of alleles identified in our screen. (The single non-null allele of *mec-3* we identified is a non-coding mutation that affects the expression pattern of the gene; such mutations are expected to be rare.)

Whereas MEC-10 modulates channel function, MEC-19 affects channel surface expression and counters the action of POML-1. POML-1 acts as an endoplasmic reticulum-resident chaperone for MEC-4 production and folding (Chen *et al.* 2016). In contrast, MEC-19, which is localized to the plasma membrane and, perhaps, the Golgi, reduces MEC-4 surface expression. MEC-19 is not part of MEC-4 channel complex, although it may transiently interact with MEC-4. Thus, the loss of *mec-19* activity causes TRN degeneration in *poml-1mec-4(d)* animals likely by increasing the number of MEC-4(d)-containing channels on the surface of the TRNs. The mechanism of MEC-19 action on the MEC-4 channel remains to be studied, in part, at least because MEC-19 is a novel protein we could find only in *Caenorhabditis* species. Given the localization of MEC-19 on the plasma membrane and its negative effect on MEC-4 surface expression, one possible hypothesis is that it may regulate the removal of the transduction channel from the plasma membrane. Alternatively, MEC-19 could inhibit the insertion of channel into the membrane. Although MEC-19 has not been found in other species, a similar mechanism may exist for other membrane proteins.

Our screen identified two genes that generated *mec-4(d)* deaths in the *poml-1* background, and the protein products of these genes normally restrict the action of MEC-4(d). By screening F2 progeny from P0 animals, we biased the screen for mutations with very strong effects. Weaker suppression of *poml-1* or enhancement of *mec-4(d)* might be revealed by testing specific candidates, such as the genes that are expressed in the TRNs, but whose loss does not produce touch insensitivity (Topalidou and Chalfie 2011). Testing the effect of RNAi for these genes on TRN cell death in *poml-1mec-4(d)* animals may identify more components that restrict *mec-4(d)* toxicity.

## 

## Supplementary Material

Supporting Information
