# Supplementary material for: MEC-10 and MEC-19 Reduce the Neurotoxicity of the MEC-4(d) DEG/ENaC Channel in Caenorhabditis elegans
Source: G3 (Bethesda). 2016 Feb 17;6(4):1121–30. doi: 10.1534/g3.115.023507 (PMC4825646; doi:10.1534/g3.115.023507)
Supplement: Supporting Information [file supp_6_4_1121__index.html]

Supporting Information 

# MEC-10 and MEC-19 Reduce the Neurotoxicity of the MEC-4(d) DEG/ENaC Channel in *Caenorhabditis elegans*

## Supporting Information for Chen *et al.*, 2016

**Files in this Data Supplement:**

- File S1 - mCherry::MEC-4 and MEC-19::EGFP on the surface of *Xenopus* oocytes. MEC-4 molecules show no apparent movement, but MEC-19 molecules are mobile. (.avi, 5,221 KB)
